# Validation of an instrument to assess the delivery of patient-centred care to people with intellectual disabilities as perceived by professionals

**DOI:** 10.1186/s12913-017-2424-8

**Published:** 2017-07-11

**Authors:** Jane Murray Cramm, Anna Petra Nieboer

**Affiliations:** Institute of Health Policy & Management (iBMG), Erasmus University, Rotterdam, The Netherlands

**Keywords:** Patient-centred care, Intellectual disability, Disability organizations, Instrument development

## Abstract

**Background:**

Patient/Person Centred Care (PCC) has achieved widespread attention which resulted in the identification of eight dimensions of PCC: Respect for the patients’ values, preferences and expressed needs; information and education; access to care; emotional support to relieve fear and anxiety; involvement of family and friends; continuity and secure transition between healthcare settings; physical comfort; coordination of care. An instrument to assess patient centeredness of care delivery according to these eight dimensions among professionals is however lacking. The main objective of this study is therefore to develop and validate an instrument to assess the eight PCC dimensions among professionals providing care to institutionalized People With Intellectual Disabilities (PWIDs).

**Methods:**

This cross-sectional survey study was conducted in a disability care centre in the region Twente in the Netherlands, the *Twentse Zorgcentra*. All professionals delivering care to institutionalized PWIDs (*n* = 1146) were invited to participate. An instrument was developed to assess the eight dimensions of PCC, which was tested among 464 professionals (response rate = 40%). We tested the instrument by means of structural equation modelling, and examined its validity and reliability.

**Results:**

Indices of the 35-item PCC version are satisfactory but showed that the model left room for improvement and shortening of the instrument (RMSEA >0.06 and CFI < 0.95). Confirmatory factor analyses revealed good indices of fit with the 24-item PCC-instrument among professionals. Internal consistency of the overall instrument was also good.

**Conclusions:**

The psychometric properties of the 24-item PCC-instrument were satisfactory, rendering it a valid and reliable instrument for assessing the eight dimensions of PCC among professionals providing care to institutionalized PWIDs.

**Electronic supplementary material:**

The online version of this article (doi:10.1186/s12913-017-2424-8) contains supplementary material, which is available to authorized users.

## Background

Ever since the Institute of Medicine (IOM) identified patient-centred care (PCC^1^) as one of the six domains of quality of care in 2001, research on this subject has grown tremendously. PCC is defined as “healthcare that establishes a partnership among practitioners, patients, and their families (when appropriate) to ensure that decisions respect patients’ wants, needs, and preferences and that patients have the education and support they need to make decisions and participate in their own care” [1]. Extensive research has identified eight dimensions of PCC: “respect for patients’ values, preferences and expressed needs”, “provision of information and education”, “access to care”, “emotional support to relieve fear and anxiety”, “involvement of family and friends”, “continuity and secure transition between healthcare settings”, “physical comfort”, and “coordination of care” [2–6]. A careful review of additional literature on PCC identified no other dimensions [7–11]. Currently, there is a lack of research on these eight PCC dimensions for people with intellectual disabilities (PWIDs). The following paragraphs provide a detailed description of the relevance of these eight dimensions in the delivery of care for PWIDs.

### Patients’ preferences

One of the core components of PCC is treating patients with dignity and respect and seeing them as whole persons, not merely seeing their disease or functional impairment. It has become increasingly important to deliver care tailored to individual needs when caring for PWIDs [12]. To do so, professionals need to be innovative, creative and motivated to identify the needs of their clients because PWIDs often experience major difficulties expressing their needs [13].

### Information and education

Another important element of PCC is the provision of complete information to patients about all aspects of their care. Information and education may help facilitate autonomy and self-care [4, 14, 15]. Due to their cognitive limitations, PWIDs require a special approach when it comes to the provision of information and education. This approach requires skill, creativity and time, which unfortunately is sometimes limited [16]. Furthermore, the level of disability should also be taken into account when designing such programs [14]. Informational leaflets especially developed for PWIDs could be beneficial in this regard [15]. Educational information may be provided in the form of pictures, symbols and simple words to enhance knowledge and skills [17].

### Access to care

Access to care includes the patients’ ability to make appointments promptly and easily and the availability of health care professionals and support for patients. For example, buildings must be accessible to all patients including those with mobility issues, clear directions must be posted in several languages including Braille for the blind and a clear, user-friendly scheduling system must be in place. Physical, social, communicational, cognitive and financial issues regarding access to care information, facilities and services either enable or inhibit inclusion and participation of PWIDs in society [18].

### Emotional support

Patients may experience anxiety about how their disabilities influence their lives in both physical and social terms. PCC requires professionals to pay attention to these types of anxiety. Due to their cognitive limitations, PWIDs experience a higher degree of information asymmetry, so they may feel more fear and anxiety when confronted with any kind of treatment or diagnostics [19]. It is especially important to provide emotional support to PWIDs as they establish social relationships, take part in social activities, develop certain skills/hobbies or engage in employment. These skills empower PWIDs by making them feel secure, strong and confidant, and they help them reduce feelings of stress, loneliness and isolation [20].

### Family and friends

Dealing with the consequences of a family member with an intellectual disability (ID) has a major effect on the entire family [21], resulting in feelings of stress and a poorer well-being [22–24]. Behavioural problems especially seem to have a negative impact on the well-being of family members [22]. Formal caregivers can provide support to the disabled family member, which alleviates some of the burden on family and friends [25]. Moreover, the well-being of parents seems to have a direct effect on the well-being of their disabled child, so investing in the well-being of a disabled person’s family and friends is also beneficial for the well-being of PWIDs [26]. Therefore, it is important to train family and friends on how to deal with the behavioural problems of a disabled person and how to cope with negative consequences in their own lives (e.g., reduced physical and social activities) [27]. PCC in this regard involves a more family-centred approach. To improve the well-being of the family, professionals need to pay attention to the needs of the family as a whole and involve them in the decision-making process [27]. Hence, the bond between patients, informal caregivers and professionals is crucial [28].

### Continuity and transition

PWIDs encounter specific problems when it comes to continuity and transition of care. Given their cognitive problems and difficulties in communication, they experience challenges expressing their symptoms, pain and medical history. Transitions during certain phases of life also seem problematic, such as the transition from paediatric to adult care [29] and transfer to elderly or nursing homes [16]. It is necessary for PWIDs to have a smooth transition led by a case manager to ensure a stable recovery. This transition includes providing clear and detailed information regarding the person’s medical history, medication use, dietary needs and other peculiarities; coordinating ongoing treatment and services after discharge or placement in a new healthcare setting; and informing patients about the possibilities of clinical, social, physical and financial support on a continuing basis [30].

### Physical comfort

PWIDs are at an increased risk of experiencing frequent or severe pain due to their high prevalence of associated disorders [16, 31]. Although self-report is the gold standard for pain measurement, it may be challenging for PWIDs due to their limited self-expression abilities, cognitive impairments and communication problems. Alternative assessment methods, such as facial impression charts, may be helpful. In addition, diverse medical and nonmedical pain management techniques could be beneficial for effective pain management in this particular population [31].

Ensuring privacy for all residents is often complicated by a lack of financial resources and a limited availability of space [16]. In recent years, much attention has been given to the living environments of PWIDs, which range from small-scale to large living facilities. Sometimes these living environments are located within a regular community or in a separate, secure environment. Attention to physical comfort, privacy and creating an appropriate atmosphere has grown in recent years [32].

### Coordination of care

All team members should be well informed so that PWIDs and/or their informal caregivers only need to tell their stories once. Patients should have a primary contact person who knows everything about their condition and treatment. Proper care coordination can reduce duplicative efforts, thus making it possible to direct the savings toward other needs [33]. Multidisciplinary collaboration is also called for. The establishment of a care plan may help improve care coordination among professionals with different occupational backgrounds who are involved in delivering care to PWIDs [16, 34].

#### Improved outcomes

There is considerable evidence on the benefits of implementing the eight PCC dimensions to achieve better organizational and patient outcomes in hospitals for the general population [2–4]; however, we lack such research on PWIDs. Enhancing the eight dimensions of PCC is expected to improve outcomes for PWIDs by addressing their needs and preferences. In addition, because PCC is designed to improve coordination among professionals as well as interactions between patients and professionals, parallel gains are expected for both patients and professionals [35]. Indeed, research suggests that PCC enhances patient care and positively affects job satisfaction by improving the working conditions of professionals [35, 36]. Research, for example, shows that professionals who perceive their work units as more patient-centred are significantly more satisfied with their jobs compared to those whose units were perceived as less patient-centred [35]. Furthermore, healthcare professionals prefer to work in organizations that promote interdisciplinary coordination and teamwork, which enhances job satisfaction [37]. As stated earlier, there is currently a lack of such studies in the field of disability research (investigating the eight dimensions of PCC and its relationship with job satisfaction).

#### Development of instruments to assess the eight dimensions of PCC

Validated instruments are needed to assess PCC. A crucial first step is developing measures to assess PCC from the perspectives of professionals. A thorough review of instruments currently used to assess PCC in PWIDs clearly indicated the need for new instruments. Therefore, the aim of this study is the development and validation of an instrument to assess the eight dimensions of PCC from the perspective of professionals providing care to institutionalized PWIDs. In this article, we describe the development and psychometric testing of a PCC instrument to assess care for PWIDs among professionals in terms of its validity and reliability.

## Methods

### Setting and participants

This cross-sectional survey study was conducted in a disability care centre in the region Twente in the Netherlands, the *Twentse Zorgcentra*. This organization is the largest provider of care for persons with different forms of ID in this region. Various forms of 24-h care and services are being provided to the clients of the center in living facilities ranging from a small- to a large-scale. All professionals working at least for a year at the *Twentse Zorgcentra* for 16 h a week at minimum involved in the care for PWIDs needing 24 h care a day were asked to fill in a questionnaire. Since we investigated professionals providing care to PWIDs only and this concerned an investigation of their experiences with care delivery (no intervention took place) approval of the research ethics committee was not needed [38]. All participants consented to participate.

### Data collection

This study included professionals involved in the care and support of clients with intellectual disabilities who required 24-h care. Only employees with permanent or temporary contracts for at least 16 h of work per week, who had worked for the organisation for at least 1 year, were selected to fill in a questionnaire, which led to a total of 1146 professionals. Data collection took place between April and June 2015 by means of postal questionnaires. In April 2015, a package was sent per mail to all 1146 professionals that included an invitation letter, a questionnaire and a return envelope. In the invitation letter, information was provided about the purpose of the study, the voluntary nature of the study as well as about the organization and researchers that were in charge of the study. Also, information was provided to ensure confidentiality. After approximately three weeks, a reminder letter was sent to all non-respondents. This strategy led to a final response of 466 professionals (40% response rate) to this survey. Two respondents only filled in background characteristics leaving PCC items with 100% missing and therefore these two respondents were eliminated from the analyses bringing the total n to 464.

### Survey instruments

#### Development of items to assess PCC

The eight dimensions of PCC as identified by the Picker Institute were used as framework to develop the items to assess PCC for PWIDs. The development of items to assess level of PCC for PWIDs builds upon earlier work [5, 6]. In these studies PCC viewpoints were identified using Q-methodology (a combination of qualitative and quantitative research) for which 35 statements were developed to assess the importance of the eight PCC dimensions. Using Q-methodology participants were given a set of statements about the PCC dimensions and instructed to rank these statements according to their level of agreement. With this methodology participants reveal their subjective viewpoints on PCC by ordering the statements. These 35 statements formed the basis for the development of the 35-item PCC questionnaire for the current study, a collaboration between the research team members as well as various professionals from the *Twentse Zorgcentra* (see Additional file 1 for a full overview of the 35-items to assess PCC for PWIDs among professionals). Responses of professionals were measured on a five-point scale ranging from 1 (never) to 5 (always), with higher mean scores indicating better PCC.

#### Job satisfaction

Since research indicates that professionals who perceive their work as more patient-centred are significantly more satisfied with their jobs compared to those whose units were perceived as less patient-centred [35], we expect that higher levels of PCC positively affect levels of job satisfaction among professionals. Therefore, we tested construct validity with the Measurement of Job Satisfaction (MJS) questionnaire [39]. Results from a systematic review showed that this instrument was the most reliable and valid instrument to assess job satisfaction [40]. The MJS measures five work factors: personnel satisfaction; workload; professional support; salary; and prospects and training. Scores on the five subscales may be summed to yield an overall job satisfaction score. Respondents were asked to rate their level of agreement on a 5-point scale ranging from 1 (very unsatisfied) to 5 (very satisfied). In this study, the Cronbach’s alpha of this instrument was 0.93 (based on all items together).

#### Background characteristics

Professionals were asked about their age, gender, educational level, marital status. In addition we asked for occupation, number of years working at the *Twentse Zorgcentra* and number of hours per week working at the *Twentse Zorgcentra.*

### Analysis

Our analyses involved the following seven steps.

1. The sample characteristics were analyzed using descriptive statistics.
2. We data-screened the items by examining the number of missing and the mean and standard deviation of each item.
3. To verify the factor structure of the 35-item questionnaires we executed confirmatory factor analysis using the LISREL program [41]. We treated the data as ordinal and used robust DWLS estimation with polychoric correlations to fit factor models. The robust DWLS method has been recommended by others [42] for ordinal data with 5 or less categories.
4. To test the measurement models, we used multiple imputation techniques using Expected Maximization algorithm. We used the following indices of model fit whose cut-off criteria were proposed by Hu and Bentler [43]:

- The overall test of goodness-of-fit assesses the discrepancy between the model implied and the sample covariance matrix via a normal-theory weighted least squares test. A plausible model has low, preferably non-significant χ^2^ values. Chi-square is, however, known to be overly sensitive when the sample size is large (more than 200 respondents) [44], resulting in difficulties to obtain the desired non-significant level [45].
- The Standardized Root Means square Residual (SRMR), which is a scale-invariant index for global fit ranging between 0 and 1. SRMR values below 0.08 indicate a good fit.
- The Root Mean Square Error of Approximation (RMSEA), which is preferably ≤0.06.
- The Comparative Fit Index (CFI), which compares the independent model (i.e., observed variables are unrelated) to the estimated model. CFI values are preferably larger than 0.95.

5. Internal consistency of the subscales was assessed by calculating Cronbach’s alphas. In addition, we investigated inter-correlations to verify conceptually relatedness among (sub)scales. We also computed a composite reliability index based on the factor loadings of the first-order constructs to assess overall scale reliability.
6. We then investigated construct validity of the instrument by analyzing the associations between the PCC instrument (total and the eight dimensions) and job satisfaction. In addition we tested the total PCC instrument with the five work factors: personnel satisfaction; workload; professional support; salary; and prospects and training. We expect to find stronger relationships between PCC, professional support and personal satisfaction for example compared to the relationship between PCC, prospects and training and salary. Job satisfaction refers to the perception that one’s job fulfils or allows the fulfillment of one’s important job values, providing and to the degree that those values are congruent with one’s needs. Therefore, we expect job satisfaction to be affected by the (in)ability of professionals to deliver patient-centered care to PWIDs. The concepts professional support and PCC (e.g. coordination of care and continuity and transition) are more related compared to salary, prospect and training.

## Results

### Sample characteristics of professionals

Table 1 displays characteristics of the professionals. The majority of the professionals that filled in the baseline questionnaire were female (86.8%). Mean age was 42.81 years (sd 11.59) ranging from 14 to 65. The majority of the professionals (87.5%) had been working for more than 5 years within the organisation. Furthermore, 70.3% of the professionals worked more than 22 h per week. The respondents mainly consisted of general support workers (33.3%), personal support workers (26.8%) and personal support workers day-care (17.4%).

**Table 1:** Sample characteristics professionals (*n* = 464)

		Percentage
Gender	- Female	86.8%
Working past	- More than 5 years	87.5%
Working hours per week	- More than 22 h per week	70.3%
Occupation	- General support worker	33.3%
	- Personal support worker	26.8%
	- Personal support worker day-care	17.4%
	- Paramedical	2.6%
	- Behavioral specialist	2.1%
	- Assistant support worker	1.7%
	- Assistant support worker day-care	1.5%
	- Others	6.5%

### Datascreening

Table 2 shows the mean, standard deviation and the number of missing responses on each PCC item. These results indicate a relatively high score (>4.0) on items ‘Healthcare professionals treat clients with dignity and respect’ , ‘Healthcare professionals work as a team in care delivery to clients’ , ‘The building is accessible to all clients’ , ‘Healthcare professionals involve relatives in decisions regarding the patient’s care’ and a low score (<2.0) on ‘Accommodation for relatives is provided’. All items had less than 20% missing responses. Data screening information was taken into account in the stepwise procedure of the item reduction analysis.

**Table 2:** Item characteristics of the first full model using all 35 PCC items (*n* = 464)

Item		Valid n	Missing	Mean	sd
*Patients’ preferences*					
**1.**	**Healthcare professionals treat clients with dignity and respect**	463	1(0.2%)	4.31	.66
**2.**	**Healthcare is focused on improving the quality of life of clients**	463	1(0.2%)	3.91	.83
**3.**	**Healthcare professionals take client’s preferences into account**	463	1(0.2%)	3.91	.71
4.	Healthcare professionals involve clients in decisions regarding their care	460	4(0.9%)	3.21	1.02
5.	Clients are supported to set and achieve their own treatment goals	456	8(1.7%)	3.18	1.12
*Physical comfort*					
**6.**	**Healthcare professionals pay attention to pain management**	453	11(2.4%)	3.50	1.14
**7.**	**Healthcare professionals take client’s preferences for support with their daily living needs into account**	462	2(0.4%)	3.90	.86
8.	Clients’ areas are clean and comfortable	441	23(5.0%)	3.19	.91
**9.**	**Clients have privacy**	460	4(0.9%)	3.29	1.02
*Coordination of care*					
**10.**	**Healthcare professionals are well-informed; clients need to tell their story only once**	453	11(2.4%)	3.31	.84
**11.**	**Care is well-coordinated between professionals**	464	0 (0.0%)	3.50	.82
12.	Clients know who is coordinating their care	451	13(2.8%)	3.65	1.17
13.	Clients have a first point of contact who knows everything about their condition and treatment	459	5(1.1%)	3.94	1.04
**14.**	**Healthcare professionals work as a team in care delivery to clients**	462	2(0.4%)	4.10	.87
*Emotional support*					
**15.**	**Healthcare professionals pay attention to client’s anxiety about their situation**	421	43(9.3%)	3.99	.88
**16.**	**Healthcare professionals involve relatives in the emotional support of the client**	456	8(1.7%)	3.61	.96
**17.**	**Healthcare professionals pay attention to client’s anxiety over the impact of their illness on their loved ones (if applicable)**	375	89(19.2%)	3.30	1.16
*Access to care*					
**18.**	**The building is accessible to all clients**	458	6(1.3%)	4.03	1.00
**19.**	**Clear directions are provided to and inside the building**	447	17(3.7)	3.16	1.25
**20.**	**It is easy to schedule an appointment**	461	3(0.6%)	3.28	1.01
21.	Waiting times for an appointment are acceptable	457	7(1.5%)	3.25	.95
22.	Language is not a barrier for access to care	453	11(2.4%)	2.93	1.28
*Continuity and transition*					
**23.**	**When a client is transferred to another ward, relevant patient information is transferred as well**	459	5(1.1%)	3.33	1.04
**24.**	**Clients who are transferred are well-informed about where they are going, what care they will receive and who will be their contact person**	440	24(5.2%)	3.55	1.00
**25.**	**Clients get skilled advice about care and support at home after discharge**	432	32(6.9%)	3.46	1.03
*Information and education*					
26.	Clients are well-informed about all aspects of their care	435	29(6.3%)	3.21	.98
**27.**	**Clients can access their care records**	406	58(12.5%)	2.87	1.47
**28.**	**Clients are in charge of their own care**	444	20(4.3%)	2.93	1.10
**29.**	**Healthcare professionals support clients to be in charge of their care**	451	13(2.8%)	3.41	1.02
30.	There is open communication between clients and healthcare professionals	449	15(3.2%)	3.83	.94
31.	Healthcare professionals have good communication skills	464	0 (0.0%)	3.72	.77
*Family and friends*					
32.	Accommodation for relatives is provided	413	51(11.0%)	1.43	.81
**33.**	**Healthcare professionals involve relatives in decisions regarding the patient’s care**	462	2(0.4%)	4.11	.91
**34.**	**Healthcare professionals pay attention to loved ones in their role as carer for the client**	461	3(0.6%)	3.91	.89
**35.**	**Healthcare professionals pay attention to the needs of family and friends of the client**	455	9(1.9%)	3.66	.92

Items in bold are included in the short version

### Confirmatory factor analysis with 35 items

Indices of the 35-item PCC version are satisfactory but showed that the model left room for improvement and shortening of the instrument (RMSEA >0.06; see Table 3).

**Table 3:** Model fit of the full 35-item PCC instrument and the short 24-item version

Multiple imputed data using EM algorithm (*n* = 464)	Χ^2^	RMSEA	CFI	SRMR
Model 1: 35 items	3465.828 (*P* = 0.0)	0.0719	0.957	0.0715
Model 2: final short version 24 items	933.335 (*P* = 0.0)	0.0474	0.986	0.0487

### Confirmatory factor analysis with 24 items

Following the factor loadings, modification indices and checking the internal consistency of each subscale, the stepwise procedure resulted in elimination of items: 4, 5 8, 12, 13, 21, 22, 26, 30, 31, and 32. The final short version consisted of 24-items with three items for each subscale. The significant Normal Theory Weighted Least Square χ^2^ statistic is not surprising given its sensitivity to sample size. All other indices indicated that the model showed a good fit [43]. When we look at the results CFI value of the 24-item PCC-instrument was above cut-off value of 0.95, the SRMR below the cut-off value of 0.08 and RMSEA ≤0.06 (Table 3). All items had factor loadings above 0.40 on the intended factor (based on single factor loadings; see Table 4).

**Table 4:** Factor loadings 24-item patient centered care instrument

Item	λ	Estimates (standard error)
*Patients’ preferences*		
1. Healthcare professionals treat clients with dignity and respect	0.771	0.771 (0.047)
2. Healthcare is focused on improving the quality of life of clients	0.689	0.689 (0.042)
3. Healthcare professionals take client’s preferences into account	0.762	0.762 (0.041)
*Physical comfort*		
4. Healthcare professionals pay attention to pain management	0.476	0.476 (0.045)
5. Healthcare professionals take client’s preferences for support with their daily living needs into account	0.699	0.699 (0.041)
6. Clients have privacy	0.537	0.537 (0.040)
*Coordination of care*		
7. Healthcare professionals are well-informed; clients need to tell their story only once	0.738	0.738 (0.046)
8. Care is well-coordinated between professionals	0.787	0.787 (0.036)
9. Healthcare professionals work as a team in care delivery to clients	0.647	0.647 (0.043)
*Emotional support*		
10. Healthcare professionals pay attention to client’s anxiety about their situation	0.764	0.764 (0.038)
11. Healthcare professionals involve relatives in the emotional support of the client	0.863	0.863 (0.029)
12. Healthcare professionals pay attention to client’s anxiety over the impact of their illness on their loved ones (if applicable)	0.789	0.789 (0.031)
*Access to care*		
13. The building is accessible to all clients	0.649	0.649 (0.053)
14. Clear directions are provided to and inside the building	0.555	0.555 (0.045)
15. It is easy to schedule an appointment	0.645	0.645 (0.041)
*Continuity and transition*		
16. When a client is transferred to another ward, relevant patient information is transferred as well	0.617	0.617 (0.045)
17. Clients who are transferred are well-informed about where they are going, what care they will receive and who will be their contact person	0.857	0.857 (0.026)
18. Clients get skilled advice about care and support at home after discharge	0.838	0.838 (0.031)
*Information and education*		
19. Clients can access their care records	0.598	0.598 (0.050)
20. Clients are in charge of their own care	0.859	0.859 (0.042)
21. Healthcare professionals support clients to be in charge of their care	0.842	0.842 (0.045)
*Family and friends*		
22. Healthcare professionals involve relatives in decisions regarding the patient’s care	0.845	0.845 (0.042)
23. Healthcare professionals pay attention to loved ones in their role as carer for the client	0.901	0.901 (0.026)
24. Healthcare professionals pay attention to the needs of family and friends of the client	0.810	0.810 (0.043)

λ = single factor loadings on the intended dimensions. All factor loadings had *p*-values < .001. Results are based on imputed data using EM Algorithm (*n* = 464)

In addition, we tested a second-order factor structure. The second-order solution also showed a good model fit (CFI = 0.98; RMSEA = 0.053; and SRMR = 0.060) and all factor loadings of the second-order factor were above .60 (again all *p* < .001). We did not formally compare both models using a χ^2^ difference test, because χ^2^ fit statistics and its derived difference test are highly sensitive to sample size. Rather, we compared the alternative goodness-of-fit indices RMSEA, CFI and SRMR. The results were comparable, although the second-order model did have a somewhat higher RMSEA value (0.053 versus 0.048).

### Internal consistency and inter-correlations

Internal consistency of the 24-item PCC-instrument as represented by a composite reliability index based on the factor loadings of the first-order constructs, yielded a value of 0.93. Internal consistency of the subscales ranged from 0.52 for the ‘physical comfort’ subscale to 0.85 for the ‘family and friends’ subscale (Table 5). Also the correlations between the full instrument and subscales are good; all (sub)scales were significantly (all *p* ≤ 0.001) and positively correlated, indicating conceptually related (sub)scales.

**Table 5:** Scale characteristics and (inter)correlations of the 24-item PCC instrument

	24-item short version	Cronbach’s alpha	Scale mean (sd)	Correlations range	1	2	3	4	5	6	7	8
1. Patients’ preferences	1, 2, 3	.71	4.05 (.58)	.31–.55								
2. Physical comfort	6, 7, 9	.52	3.57 (.72)	.40–.56	.55***							
3. Coordination of care	10, 11, 14	.69	3.64 (.66)	.32–.55	.55***	.54***						
4. Emotional support	15, 16, 17	.77	3.63 (.76)	.34–.56	.45***	.56***	.43***					
5. Access to care	18, 19, 20	.59	3.49 (.76)	.28–.45	.40***	.41***	.45***	.34***				
6. Continuity and transition	23, 24, 25	.77	3.44 (.82)	.44–.36	.44***	.40***	.44***	.42***	.44***			
7. Information and education	27, 28, 29	.74	3.07 (.94)	.31–.41	.31***	.41***	.32***	.41***	.31***	.41***		
8. Family and friends	33, 34, 35	.85	3.89 (.79)	.28–.54	.49***	.46***	.41***	.54***	.28***	.36***	.33***	
9. Overall PCC	All of the above	.93^a^	3.60 (.53)	.65–.76	.72***	.76***	.71***	.74***	.65***	.70***	.66***	.69***

***p < 0.001 (2-tailed). Results using the 35-item version are similar. Results are based on listwise deletion of missing cases

^a^Composite reliability index based on the factor loadings of the first-order construct

### Construct validity

Construct validity was investigated by assessing the relationship between PCC and job satisfaction (Table 6). Results show that all eight dimensions of PCC are positively related to job satisfaction (all *p* ≤ 0.001). These values indicated construct validity. Table 7 shows the relationship between PCC and the subscales of job satisfaction: Personnel satisfaction, workload, professional support, salary and prospects and training.

**Table 6:** Correlation analyses of the PCC dimensions (the 24-item version) with job satisfaction

	Job satisfaction
Patients’ preferences	.34***
Physical comfort	.30***
Coordination of care	.29***
Emotional support	.20***
Access to care	.31***
Continuity and transition	.32***
Information and education	.18***
Family and friends	.19***
Overall PCC	.38***

***p < 0.001 (2-tailed). Results using the 35-item version are similar. Results are based on listwise deletion of missing cases

**Table 7:** Correlation analyses of the PCC dimensions (the 24-item version) with job satisfaction

	Overall PCC
Personnel satisfaction	.37***
Workload	.20***
Professional support	.40***
Salary	.24***
Prospects and training	.24***

***p < 0.001 (2-tailed). Results using the 35-item version are similar. Results are based on listwise deletion of missing cases

## Discussion

This study aimed to develop and validate an instrument to assess the eight dimensions of PCC from the perspective of professionals providing care to institutionalized PWIDs. Our results showed that the 24-item PCC-instrument is a valid and reliable instrument to measure PCC and its eight dimensions from the perspectives of professionals working with PWIDs.

The advantages achieved by healthcare organizations delivering high-level PCC are likely to enhance job satisfaction among their employees. Looking at the strength of the relationship delivering care in accordance with patients’ preferences shows the strongest relationship with job satisfaction, while educating patients and providing them with information shows the weakest relationship with job satisfaction. As expected, stronger relationships were found with professional support and personal satisfaction and weaker relationships with training, salary and workload. The benefits of implementing the eight PCC dimensions to achieve better organizational and patient outcomes in hospitals for the general population were already known [2–4]. This is the first research showing that organizations for PWIDs aiming to improve job satisfaction among their employees benefit from making their organizations more patient-centered. Especially the ability of professionals to deliver care that fits the needs and preferences of PWIDs improves job satisfaction, which is also known to improve outcomes for PWIDs. With the integration of the eight interrelated PCC dimensions, the system is reformed such that proactive professional teams can co-create care delivery together with PWIDs from which they both benefit. Not being able to deliver care that meets the needs and preferences of PWIDs, in turn, is known to cause stress, frustrates professionals and instigates behavioral problems among clients [46, 47] and potentially harms quality of care available for clients [48]. PCC as such may act as a resource of work support and personal satisfaction, which are known to lessen feelings of hopelessness, stress and depression among professionals working with PWIDs [49]. Investing in patient-centeredness is, therefore, expected to improve outcomes for clients as well as professionals working with them.

This study comes with limitations. This study, for example, did not include the predictive value of the 24-item PCC-instrument. Future research is needed to assess the instrument’s sensitivity to change. We also recommend testing the English version of the 24-item PCC-instrument in other countries to ensure international validity. Using the 24-item PCC-instrument to investigate PCC in other type of organizations and settings (eg primary care, hospitals) is also called for. Looking at the Cronbach’s alphas of the 24-item PCC-instrument subscales most of them indicate reliability. The subscales physical comfort and access to care are, however, below 0.60 (0.52 and 0.59 respectively). Looking at the underlying items of physical comfort, this may not be surprising since conceptually privacy lies a bit further apart from pain management for example. This also applies to the access to care items; setting up an appointment may be experienced very differently from accessibility of the building and the directions provided to and inside the building.

The high value of 0.93 for the composite reliability index suggests that overall reliability of the instrument is good. This is also supported by the second-order structure we found, supporting that the subscales can be accounted for by one underlying higher-order construct. Therefore, we feel this is a promising instrument to assess PCC among professionals providing care to PWIDs. Finally, we did not include the perceptions of PWIDs. Future research is necessary to develop and validate an instrument to assess patient-centeredness in organizations from their perspective. Since earlier research did show that professionals’ perceptions of care quality and changes therein predict more positive experiences of patients with care delivery over time [50] we feel that an instrument assessing PCC among professionals is of added value.

## Conclusions

We conclude that the psychometric properties of the 24-item PCC-instrument to assess levels of PCC provided to PWIDs among professionals are good and that the PCC instrument is promising to assess the eight dimensions of PCC provided to PWIDs in disability organizations.

## Additional file

Additional file 1: Appendix patient centred care questionnaire. Measurement instrument to assess the eight dimensions of PCC. The 35 items of the questionnaire representing the eight dimensions of PCC. (DOCX 14 kb)

## Abbreviations

PCC: Patient/Person centred care
PWIDs: People with intellectual disabilities

## Footnotes

1 Those receiving care are often referred to as “clients”, “patients”, “persons”, or “residents”. In this research, the term “patients” is considered to encompass these meanings; thus, “patient-centred care” is considered to encompass “resident-centred care”, “person-centred care”, and “client-centred care”.
