# Supplementary material for: Validation of an instrument to assess the delivery of patient-centred care to people with intellectual disabilities as perceived by professionals
Source: BMC Health Serv Res. 2017 Jul 11;17:472. doi: 10.1186/s12913-017-2424-8 (PMC5504747; doi:10.1186/s12913-017-2424-8)
Supplement: Additional file 1: — Appendix patient centred care questionnaire. Measurement instrument to assess the eight dimensions of PCC. The 35 items of the questionnaire representing the eight dimensions of PCC. (DOCX 14 kb) [file 12913_2017_2424_MOESM1_ESM.docx]

**APPENDIX PATIENT CENTRED CARE QUESTIONNAIRE**

| **PCC Dimensions** | **Questions** | **Scoring 1 - 5** |
| --- | --- | --- |
| Patients’ preferences | 1. Healthcare professionals treat clients with dignity and respect. | 5. always  4. often  3. regularly  2. sometimes  1. never |
|  | 2. Healthcare is focused on improving the quality of life of clients. | 5. always  4. often  3. regularly  2. sometimes  1. never |
|  | 3. Healthcare professionals take client's preferences into account. | 5. always  4. often  3. regularly  2. sometimes  1. never |
|  | 4. Healthcare professionals involve clients in decisions regarding their care. | 5. always  4. often  3. regularly  2. sometimes  1. never |
|  | 5. Clients are supported to set and achieve their own treatment goals. | 5. always  4. often  3. regularly  2. sometimes  1. never |
| Physical comfort | 6. Healthcare professionals pay attention to pain management. | 5. always  4. often  3. regularly  2. sometimes  1. never |
|  | 7. Healthcare professionals take client's preferences for support with their daily living needs into account. | 5. always  4. often  3. regularly  2. sometimes  1. never |
|  | 8. Areas are clean and comfortable. | 5. always  4. often  3. regularly  2. sometimes  1. never |
|  | 9. Clients have privacy. | 5. always  4. often  3. regularly  2. sometimes  1. never |
| Coordination of care | 10. Healthcare professionals are well-informed; clients need to tell their story only once. | 5. always  4. often  3. regularly  2. sometimes  1. never |
|  | 11. Care is well-coordinated between professionals. | 5. always  4. often  3. regularly  2. sometimes  1. never |
|  | 12. Clients know who is coordinating their care. | 5. always  4. often  3. regularly  2. sometimes  1. never |
|  | 13. Clients have a first point of contact who knows everything about their condition and treatment. | 5. always  4. often  3. regularly  2. sometimes  1. never |
|  | 14. Healthcare professionals work as a team in care delivery to clients. | 5. always  4. often  3. regularly  2. sometimes  1. never |
| Emotional support | 15. Healthcare professionals pay attention to client's anxiety about their situation. | 5. always  4. often  3. regularly  2. sometimes  1. never |
|  | 16. Healthcare professionals involve relatives in the emotional support of the client. | 5. always  4. often  3. regularly  2. sometimes  1. never |
|  | 17. Healthcare professionals pay attention to client's anxiety over the impact of their illness on their loved ones. | 5. always  4. often  3. regularly  2. sometimes  1. never |
| Access to care | 18. The building is accessible to all clients. | 5. always  4. often  3. regularly  2. sometimes  1. never |
|  | 19. Clear directions are provided to and inside the building. | 5. always  4. often  3. regularly  2. sometimes  1. never |
|  | 20. It is easy to schedule an appointment. | 5. always  4. often  3. regularly  2. sometimes  1. never |
|  | 21. Waiting times for an appointment are acceptable. | 5. always  4. often  3. regularly  2. sometimes  1. never |
|  | 22. Language is not a barrier for access to care. | 5. always  4. often  3. regularly  2. sometimes  1. never |
| Continuity and transition | 23. When a client is transferred to another ward, relevant patient information is transferred as well. | 5. always  4. often  3. regularly  2. sometimes  1. never |
|  | 24. Clients who are transferred are well-informed about where they are going, what care they will receive and who will be their contact person. | 5. always  4. often  3. regularly  2. sometimes  1. never |
|  | 25. Clients get skilled advice about care and support at home after discharge. | 5. always  4. often  3. regularly  2. sometimes  1. never |
| Information and education | 26. Clients are well-informed about all aspects of their care. | 5. always  4. often  3. regularly  2. sometimes  1. never |
|  | 27. Clients can access their care records. | 5. always  4. often  3. regularly  2. sometimes  1. never |
|  | 28. Clients are in charge of their own care. | 5. always  4. often  3. regularly  2. sometimes  1. never |
|  | 29. Healthcare professionals support clients to be in charge of their care. | 5. always  4. often  3. regularly  2. sometimes  1. never |
|  | 30. There is open communication between clients and healthcare professionals. | 5. always  4. often  3. regularly  2. sometimes  1. never |
|  | 31. Healthcare professionals have good communication skills. | 5. always  4. often  3. regularly  2. sometimes  1. never |
| Family and friends | 32. Accommodation for relatives is provided. | 5. always  4. often  3. regularly  2. sometimes  1. never |
|  | 33. Healthcare professionals involve relatives in decisions regarding the patient’s care. | 5. always  4. often  3. regularly  2. sometimes  1. never |
|  | 34. Healthcare professionals pay attention to loved ones in their role as carer for the client. | 5. always  4. often  3. regularly  2. sometimes  1. never |
|  | 35. Healthcare professionals pay attention to the needs of family and friends of the client. | 5. always  4. often  3. regularly  2. sometimes  1. never |
